# Estimation of the Corneal Young's Modulus *In Vivo* Based on a Fluid-Filled Spherical-Shell Model with Scheimpflug Imaging

**DOI:** 10.1155/2017/5410143

**Published:** 2017-11-08

**Authors:** Po-Jen Shih, Chun-Ju Huang, Tzu-Han Huang, Hung-Chou Lin, Jia-Yush Yen, I-Jong Wang, Hui-Jyun Cao, Wen-Pin Shih, Chi-An Dai

**Affiliations:** ^1^Department of Civil and Environmental Engineering, National University of Kaohsiung, Kaohsiung, Taiwan; ^2^Department of Mechanical Engineering, National Taiwan University, Taipei, Taiwan; ^3^Dr. Lin's Eye Clinic and Laser Vision Correction Center, Taoyuan City, Taiwan; ^4^Department of Ophthalmology, College of Medicine, National Taiwan University, Taipei, Taiwan; ^5^Department of Chemical Engineering, National Taiwan University, Taipei, Taiwan

## Abstract

Current intraocular pressure (IOP) measurement using air puff could be erroneous without applying proper corrections. Although noncontact tonometry is not considered to be accurate, it is still popularly used by eye clinics. It is thus necessary to extract the correct information from their results. This study proposes a practical approach to correctly measure IOP *in vivo*. By embedding a new model-based correction to the Corvis® ST, we can extract the corneal Young's modulus from the patient data. This Young's modulus can be used to correct the IOP readings. The tests were applied to 536 right eyes of 536 healthy subjects (228 male and 308 female) between March of 2012 and April of 2016. The tests were applied to patients at the Department of Ophthalmology, National Taiwan University Hospital and the Hung-Chuo Eye Clinics. The statistical analysis showed that the value for the Young's modulus was independent of all the other parameters collected from the Corvis ST, including the corneal thickness and the intraocular pressure. Therefore, it is important to independently measure the Young's modulus instead of depending on the correlation with the other parameters. This study adds the methodology of measuring corneal stiffness *in vivo* for ophthalmologists' reference in diagnosis.

## 1. Introduction

The biomechanical properties of the cornea are associated with the development of corneal diseases such as keratoconus, ectasia after refractive surgery, and possible glaucoma progression [1–4]. Hence, there has been a recent surge of interest in assessing corneal biomechanical properties due to potential clinical implications [5–7]. Moreover, the biomechanical properties of the cornea have been proposed to directly affect intraocular pressure (IOP) measurements, especially for normal tension glaucoma [8, 9], and are becoming recognized as necessary for the calibration of IOP in different moduli of tonometers [10–13]. Modern *in vivo* instruments such as the Ocular Response Analyzer (ORA; Reichert Ophthalmic Instruments, Buffalo, NY, USA) and Corvis® ST (Oculus, Wetzlar, Germany) not only provide their own biomechanical parameters and indices of the cornea [14, 15] but also provide the corrected IOP based on these parameters [16, 17]. The indices are useful for measuring corneal biomechanical properties. For example, the ORA provides the corneal hysteresis (CH) and the corneal resistance factor (CRF) [18] and the Corvis ST provides the deformation amplitude (DA) and the first applanation (A1) time [19]. However, the corneal biomechanical parameters derived from these instruments are not independent parameters, and they are also affected by the corneal thickness, IOP, and corneal geometry. In many cases, these parameters cannot differentiate the subclinical ectatic corneas from normal corneas [20]. Furthermore, these indicators cannot be translated into the commonly used mechanical properties such as the Young's modulus in particular [21]. The Young's modulus is an accepted biomechanical property [22] that may be used for predicting postrefractive surgery ectasia [23, 24], detecting early keratoconus [6, 25], and reshaping orthokeratology [23, 26, 27]. It is highly desirable to find means to extract the Young's modulus, which is independent of the corneal thickness and other parameters, from *in vivo* measurements for clinical implementation.

The Young's modulus is known to be an important parameter in clinical practices and researches [6, 28–30], and there have been many methods trying to measure the Young's modulus. Buzard and Torres et al. established the corneal biomechanical models correlating the force and displacement measurement from the tonometer *in vivo* [31, 32]. Despite the efforts, the problem remains that most of the experimental measurements were performed on cadaver eyes. The clinically applicable procedures were still lacking. Furthermore, the Young's moduli reported in the literature were within the range of 0.1–10 MPa from *in vitro* tests (listed in Table S1 available online at https://doi.org/10.1155/2017/5410143). Importantly, the Young's modulus derived from an *in vivo* measurement might be potentially different from that from an *in vitro* measurement.

The current research used the Scheimpflug images captured by the high-speed camera (4330 frames/sec) in the Corvis ST to extract the corneal Young's modulus *in vivo*. The Scheimpflug images illustrated corneal deformation. Using image processing techniques, it is possible to extract the dynamic behavior of the cornea by the air puff [33]. Our model refers to several previous studies. For example, Kling et al. fit finite element analysis results with the Corvis ST and then used an inverse model to find the biomechanical parameters [34]. We also note that most of the previous approaches either used time sequences with optimal fitting or complex numerical approaches, both of which are time-consuming and are inherently offline calculations. Readers are referred to Garcia-Porta et al. for a review of the corneal biomechanical properties measurement techniques [35].

Our study aims to establish an analytical solution for a more realistic model that enables the extraction of the Young's modulus *in vivo*. By using Taber's shell model, which describes the static deformation of a fluid-filled shell subject to a concentrated load, we were able to consider the large corneal deflection under air puff [36, 37]. We developed an image processing tool that automatically extracted the necessary geometrical information from the high-speed images. We then developed a novel formula for the calculation of the corneal Young's modulus using the information extracted from the corneal deformation. This data could be a useful information for the ophthalmologist's reference in their diagnosis. The proposed formula could be easily incorporated into the present day digital tonometer and deduce *in vivo* the corneal biomechanical parameters.

## 2. Materials and Methods

### 2.1. Mathematical Model: The Modified Taber's Model

We propose a modified Taber's model [36] to describe the relationship between the applied force and the corneal deflection. Briefly, three equilibrium factors, including the external applied force, the internal fluid pressure, and the shell stiffness force, are considered as the fluid-filled spherical shell undergoes large deformations. The following assumptions are proposed for the closed-form solution: (1) the cornea is assumed to be a portion of the hemisphere and is composed of a homogeneous, isotropic, uniform thickness, elastic material with all geometric and material properties taken as constants; (2) an incompressible fluid fills the inside of the cornea; a vertical load *P* is applied at the apex; (3) the edge of the corneal hemisphere is clamped respective to the limbus; and (4) the dynamic corneal deflection at the moment of applanation could be regarded as the static model (as the principle used in the Corvis ST).

**Figure 1:**
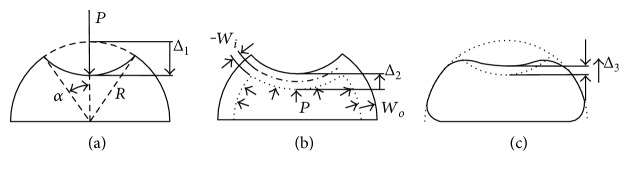
Deflection components for fluid-filled cornea: (a) dimple deflection, (b) stretching, and (c) bending.

The fluid-filled cornea modeling process proceeds in three consecutive steps: the dimple, the pressure stretching, and the bending. First, the shell is pressed down from its center and then forms a dimple, which is cut along the general meridional angle *α* and the radius of the hemispherical cornea is *R* as shown in Figure 1(a).

In this figure, the shell can be divided into two parts: the upper shell that is convex upward and the lower shell that remains concave downward. We assume a uniform bending moment around the edge, which divides the upper and lower shells. The bending moment bends down the upper shell to form the dimple. The traversal distance at the center of the dimple is thus

(1) $$\begin{array}{c} {∆}_{1}=2R\left(1-\cos\alpha \right), \end{array}$$

and the inverting dimple bends down across a volume of

(2) $$\begin{array}{c} \Delta V_{1}=\frac{-\pi R\Delta_{1}^{2}\left(6-\left(\Delta_{1}\right)/R\right)}{12}. \end{array}$$

Second, the fluid pressured *p* uniformly increases the strain throughout the lower and upper shells and causes the radial displacements *w_i_* and *w_o_*, respectively, as shown in Figure 1(b). The meridional and hoop strains are given by

(3) $$\begin{array}{c} \varepsilon_{\phi }=\varepsilon_{\theta }=\left\{\begin{array}{l} {\overset{¯}{w}}_{i}=\frac{w_{i}}{R}\;upper\;shell,\\ {\overset{¯}{w}}_{o}=\frac{w_{o}}{R}\;lower\;shell. \end{array}\right. \end{array}$$

The strain energy induced by the fluid pressure can be explained by

(4) $$\begin{array}{c} U_{S}=\frac{\pi EtR^{2}}{1-\nu^{2}}\int \left(\varepsilon_{\phi }^{2}+\varepsilon_{\theta }^{2}+2\nu \varepsilon_{\phi }\varepsilon_{\theta }\right)\sin\phi d\phi , \end{array}$$

where *E* is the Young's modulus, *t* is the shell thickness (corneal center thickness, CCT), *ν* is the Poisson's ratio, and *ϕ* is the meridional angle depending on the angles in the upper shell (0 < *ϕ* < *α*) or the lower shell (*α* < *ϕ* < *π*/2). The strain energy includes two parts: the stretching of the lower shell and the compression of the upper shell. Substituting (3) into (4), the integration yields

(5) $$\begin{array}{c} U_{S}=\frac{2\pi EtR^{2}}{1-v}\left[{\bar{w}}_{i}^{2}\left(1-\cos\alpha \right)+{\bar{w}}_{o}^{2}\;\cos\alpha \right]. \end{array}$$

The fluid pressure causes the lower shell to swell and thus further drags down the upper shell by a distance of

(6) $$\begin{array}{c} \Delta_{2}=R\left[{\bar{w}}_{i}\left(1-\cos\alpha \right)-{\bar{w}}_{o}\;\cos\alpha \right]. \end{array}$$

This subtracted volume by the downward movement, Δ_2_, of the upper shell becomes

(7) $$\begin{array}{c} \Delta V_{2}=-\Delta_{2}\int_{0}^{2\pi }\int_{0}^{\alpha }R^{2}\sin\phi \;d\;\phi \;d\theta =-2\pi R^{2}\Delta_{2}\left(1-\cos\alpha \right). \end{array}$$

In addition, the volume displaced by the inner shell is transferred to the expanding of the outer shell and adds to the volume.

(8) $$\begin{array}{c} ∆V_{3}={\bar{w}}_{o}\int_{0}^{2\pi }\int_{\frac{\pi }{2}}^{\pi -\alpha }R^{2}\sin\phi d\phi d\theta =2\pi R^{3}{\bar{w}}_{o}\;\cos\alpha . \end{array}$$

Third, the bending moments at the dimple edge and the clamped edge must be accounted for to satisfy the continuity condition in the narrow zones near the edges (as shown in Figure 1(c)). The bending strain energy for each edge is independent, and it is due to the applied edge loads.

(9) $$\begin{array}{c} U_{B}=2\pi R\sin\phi_{e}{\left[\int_{0}^{\chi }M_{\phi }d\chi +\int_{0}^{h}Hdh\right]}_{e}. \end{array}$$

In (9), *M_ϕ_* is the meridional moment, *H* is the horizontal force, *χ* is the rotation angle, and *h* is the horizontal displacement. *ϕ*_*e*_ is the meridional edge angle, where *ϕ*_*e*_ = *α* for the edge of inner shell, *ϕ*_*e*_ = *π* − *α* for the top edge of the outer shell, and *ϕ*_*e*_ = *π*/2 for the lower edge of the outer shell. Ranjan's thin shell model [38] has provided this derivation for the details of moderating the edge forces *M_ϕ_* and *H* in terms of edge displacements *χ* and *h*. After substituting the boundary conditions at these edges, the strain energy from bending is obtained:

(10) $$\begin{array}{c} U_{B}=2\pi Etc^{2}\lambda \left(\frac{\sin\alpha }{\alpha }\right)\left\{\sqrt{2}\left[\alpha^{3}+\lambda^{2}\alpha \left(y_{3}^{2}+y_{4}^{2}\right)\right]+2\lambda^{2}\alpha y_{3}y_{4}-\lambda \alpha^{2}\Delta \overset{¯}{w}+\sqrt{2}\lambda^{2}\alpha \Delta {\overset{¯}{w}}^{2}+2\lambda^{2}\alpha y_{3}y_{4}-\lambda \alpha^{2}\Delta \overset{¯}{w}+\sqrt{2}\lambda^{2}\alpha \Delta {\bar{w}}^{2}-\left(\frac{\alpha \;\cot\alpha }{10}\right)\left[2\sqrt{2}\left(\alpha^{3}+3\lambda^{2}\alpha y_{3}^{2}\right)+4\lambda^{2}\alpha y_{3}y_{4}-\lambda \alpha^{2}\Delta \overset{¯}{w}-\lambda^{3}\Delta \overset{¯}{w}\left(y_{3}^{2}+2y_{4}^{2}+\frac{\Delta {\overset{¯}{w}}^{2}}{6}\right)\right]\right\}+\lambda^{3}\sin\alpha \left[\frac{\sqrt{2}{\overset{¯}{w}}_{o}^{2}}{2}+\frac{\lambda {\overset{¯}{w}}_{o}^{3}\;\cot\alpha }{15}\right], \end{array}$$

in which $\lambda =\sqrt{R/c}$, $c=t/\sqrt{12\left(1-\nu^{2}\right)}$, $∆\bar{w}={\bar{w}}_{o}-{\bar{w}}_{i}$, and *y*_3_ and *y*_4_ are the normalized rotational angle and normalized horizontal displacement. The additional displacement induced by the bending edges can now be deduced as

(11) $$\begin{array}{c} \Delta_{4}=R\left[-2y_{4}\;\cos\alpha +\frac{\alpha }{2\sqrt{2}\;\sin\alpha }\left[2y_{3}-\frac{\lambda }{\alpha }y_{4}\Delta \bar{w}\right]-\frac{\lambda }{4\sqrt{2}}{\bar{w}}_{o}^{2}\right], \end{array}$$

and the volume change produced by Δ_3_ is

(12) $$\begin{array}{c} \Delta V_{4}=-2\pi R^{2}\Delta_{3}\left(1-\cos\alpha \right). \end{array}$$

From the above derivations, we are in a position to calculate the overall displacement. On the other hand, the work of the external force *P* inducing the total displacement change is

(13) $$\begin{array}{c} U_{P}=-P\left(\Delta_{1}+\Delta_{2}+\Delta_{4}\right), \end{array}$$

and the work of the fluid pressure *p* inducing the volume change is

(14) $$\begin{array}{c} U_{pr}=-p\left(\Delta V_{1}+\Delta V_{2}+{\Delta V}_{3}+∆V_{4}\right). \end{array}$$

Then, the total potential energy of the system is written as

(15) $$\begin{array}{c} \Pi =U_{S}+U_{B}+U_{P}+U_{pr}. \end{array}$$

After substituting the potential energy into (15), we also calculate the equilibrium by applying

(16) $$\begin{array}{c} \frac{\partial \Pi }{\partial {\bar{w}}_{i}}=\frac{\partial \Pi }{\partial {\bar{w}}_{o}}=\frac{\partial \Pi }{\partial p}=\frac{\partial \Pi }{\partial y_{3}}=\frac{\partial \Pi }{\partial y_{4}}=0. \end{array}$$

Equation (16) consists of five nonlinear algebraic equations with five unknowns: *w_i_*, *w_o_*, *p*, *y*_3_, and *y*_4_. Thus, the force-deflection relation of the eyeball could be written into a matrix identity.

(17) $$\begin{array}{c} A\cdot Z=B_{L}+B_{NL}, \end{array}$$

where the solution vector is

(18) $$\begin{array}{c} Z={\left\{{\bar{w}}_{i}\;{\bar{w}}_{o}\;\bar{p}\;y_{3}\;y_{4}\right\}}^{T}, \end{array}$$

with $\bar{p}={pR}^{2}/{Et}^{2}$ being the normalized fluid pressure. The matrix **A** is

(19) $$\begin{array}{c} A=\left[\begin{array}{cc} \frac{1}{\sqrt{2}}+\frac{2\lambda }{1-\nu }\frac{1-\cos\alpha }{\sin\alpha } & -\frac{1}{\sqrt{2}}\\ -\frac{1}{\sqrt{2}} & \frac{3}{\sqrt{2}}+\frac{2\lambda }{1-\nu }\cot\alpha \\ \begin{array}{c} -\left(1-\cos\alpha \right)\\ 0\\ 0 \end{array} & \begin{array}{c} \cos\alpha \left(2-\cos\alpha \right)\\ 0\\ 0 \end{array} \end{array}\right.\left.\begin{array}{ccc} \frac{t}{\lambda c}\frac{{\left(1-\cos\alpha \right)}^{2}}{\sin\alpha } & 0 & 0\\ -\frac{t}{\lambda c}\left(2-\cos\alpha \right)\;\cot\alpha & 0 & 0\\ \begin{array}{c} 0\\ 0\\ -\frac{t}{\lambda c}\left(1-\cos\alpha \right)\;\cot\alpha \end{array} & \begin{array}{c} 0\\ \sqrt{2}\\ 1 \end{array} & \begin{array}{c} 2\;\cos\alpha \left(1-\cos\alpha \right)\\ 1\\ \sqrt{2} \end{array} \end{array}\right]. \end{array}$$

The vector **B***_L_* represents the linear term

(20) $$\begin{array}{c} B_{L}=\left[\begin{array}{c} \begin{array}{c} -\frac{\alpha }{\lambda }+\frac{\overset{¯}{P}}{2\pi }\frac{t}{\lambda c}\frac{1-\cos\alpha }{\sin\alpha }\\ \frac{\alpha }{\lambda }-\frac{\overset{¯}{P}}{2\pi }\frac{t}{\lambda c}\cot\alpha \end{array}\\ {\left(1-\cos\alpha \right)}^{2}\\ \begin{array}{c} 0\\ -\frac{\overset{¯}{P}}{2\pi }\frac{t}{\lambda c}\cot\alpha \end{array} \end{array}\right], \end{array}$$

and the vector **B***_NL_* represents the nonlinear term

(21) BNL=λ cotα10α2λ2+y32+2y42+12Δw¯2+tcy422 sin2 αP∗−λ cotα10α2λ2+y32+2y42+12Δw¯2−tcy422 sin2 αP∗−P¯2πtλccotα−λ cotα5w¯o2−1−cosα33+α221−cosαsinα2y3−λαy4Δw¯λ cotα1062αλy3+2αλy4−y3Δw¯+tλcαP∗22 sin2 αcotα5αy3−λΔw¯y4−tcΔw¯P∗42 sin2 α,

with $P^{*}=\left(\overset{¯}{P}\right)/2\pi -\left(1-\cos\alpha \right)\overset{¯}{p}$, and $\overset{¯}{P}=P/\left(Et^{2}\right)$ being the normalized applied force.

Equation (17) represents a clean closed-form solution to the deformation of the spherical shell under the action of a point force. Unfortunately, (18)~(21) involve a few coupled high-order terms, making it very hard to solve even with numerical approximations. It is reasonable to set *y*_3_ and *y*_4_ to zero when considering clinical situations since *t*/*R* < 5% and the deflection induced by the external force is much larger than the deflections induced both by the internal pressure and the bending moment (i.e., Δ_2_, Δ_3_ ≪ Δ_1_). Thus, (20) and (21) are reduced to

(22) $$\begin{array}{c} B=B_{L}+B_{NL}=\left[\begin{array}{c} -\frac{\alpha }{\lambda }+\frac{\lambda \;\cot\alpha }{10}\left[\frac{\alpha^{2}}{\lambda^{2}}+\frac{1}{2}\Delta {\overset{¯}{w}}^{2}\right]+\frac{\overset{¯}{P}}{2\pi }\frac{t}{\lambda c}\frac{1-\cos\alpha }{\text{sin}\alpha }\\ \frac{\alpha }{\lambda }-\frac{\lambda \cot\alpha }{10}\left[\frac{\alpha^{2}}{\lambda^{2}}+\frac{1}{2}\Delta {\overset{¯}{w}}^{2}\right]-\frac{\overset{¯}{P}}{2\pi }\frac{t}{\lambda c}\cot\alpha -\frac{\lambda \;\cot\alpha }{5}{\overset{¯}{w}}_{o}^{2}\\ {\left(1-\cos\alpha \right)}^{2}-\frac{{\left(1-\cos\alpha \right)}^{3}}{3}\\ \frac{t}{\lambda c}\frac{\alpha }{2\sqrt{2}{\;\sin}^{2}\;\alpha }\left[\frac{\overset{¯}{P}}{2\pi }-\left(1-\cos\alpha \right)\overset{¯}{p}\right]\\ -\frac{\overset{¯}{P}}{2\pi }\frac{t}{\lambda c}\cot\alpha \end{array}\right]. \end{array}$$

Setting the fourth and fifth elements of **Z**, namely *y*_3_ and *y*_4_, to zero leads to

(23) $$\begin{array}{c} \bar{P}=2\pi \left(1-\cos\alpha \right)\bar{p}. \end{array}$$

### 2.2. The Simplified Model

Equation (23) describes the equilibrium condition between the external loading and the internal fluid pressure for the case of small deformations. This means that the ratio between the two forces becomes $\bar{P}/\left(2\pi R^{2}\bar{p}\right)=1-\text{cos}\alpha$. Substituting (23) into (22) to replace $\bar{P}$, and then substituting (20) into (15) to replace **B***_L_* and **B***_NL_*, we have the modified relationship

(24) $$\begin{array}{c} \left[\begin{array}{cc} \frac{1}{\sqrt{2}}+\frac{2\lambda }{1-\nu }\frac{1-\cos\alpha }{\sin\alpha } & -\frac{1}{\sqrt{2}}\\ -\frac{1}{\sqrt{2}} & \frac{3}{\sqrt{2}}+\frac{2\lambda }{1-\nu }\cot\alpha \end{array}\right]\left\{\begin{array}{c} {\overset{¯}{w}}_{i}\\ {\overset{¯}{w}}_{o} \end{array}\right\}=\left\{\begin{array}{c} -\frac{\alpha }{\lambda }+\frac{\lambda \;\cot\alpha }{10}\left[\frac{\alpha^{2}}{\lambda^{2}}+\frac{1}{2}\Delta {\overset{¯}{w}}^{2}\right]\\ \frac{\alpha }{\lambda }-\frac{\lambda \;\cot\alpha }{10}\left[\frac{\alpha^{2}}{\lambda^{2}}+\frac{1}{2}\Delta {\overset{¯}{w}}^{2}\right]+\frac{t}{\lambda c}\frac{\cos^{2}\;\alpha }{\sin\alpha }\overset{¯}{p} \end{array}\right\}. \end{array}$$

From Figure 1, the vertical displacement is defined by

(25) $$\begin{array}{c} \frac{\Delta }{R}={∆}_{1}+{∆}_{2}+{∆}_{4}=\left(2+{\overset{¯}{w}}_{i}\right)\left(1-\cos\alpha \right)-{\overset{¯}{w}}_{o}\;\cos\alpha -\frac{1}{4\sqrt{2}}{\overset{¯}{w}}_{o}^{2}. \end{array}$$

Equations (24) and (25) now provide a simplified relationship between the vertical deflection of the eyeball Δ (or ${\bar{w}}_{i}$ and ${\bar{w}}_{o}$) and the external pressure $\bar{P}$ (the applied force). Notice that the biomechanical properties such as Young's modulus and the Poisson's ratio are embedded within the coefficients. It is possible to deduce the desirable property if enough measurement data are available. In other words, it is possible to deduce the Young's modulus out of the measured geometrical deformations and the measured internal fluid pressure, which is IOP here. The calculation time for these equations is less than 10 seconds using a regular personal computer. It would be a simple matter to attach this function to a conventional tonometer.

### 2.3. The Numerical Method

Based on the proposed model in (24) and (25), the parameters required from the Corvis ST for the calculation are the IOP value *p*, the corneal radius *R*, the maximal corneal deflection Δ, the dimple edge angle *α*, and material thicknesses *t*. In this study, we assumed a static case at the moment when the maximal deflection is achieved during the air puffing process. Although the actual deflection of the cornea is a dynamic procession, we treat the brief moment as frozen and neglect the inertial force term $m\ddot{x}$ in the motion equation ($m\ddot{x}+kx=P$, in which the velocity term is zero at maximum deflection). It was further assumed that the maximal deflection occurs at the moment when the maximum air puff pressure is reached. In other words, there is no time delay in the dynamic deformation.

**Figure 2:**
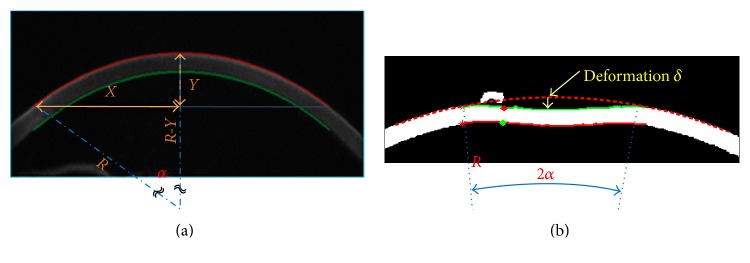
Images collected from the Corvis ST and the geometric relationship marked (a) before cornea deflection and (b) at the moment of maximum deflection.

Figure 2 shows a Scheimpflug image obtained from the test. An image processing tool developed from the MATLAB toolbox helps to automatically identify the cornea as well as the relative dimensions required. Through the series of pictures, the image processing tool automatically identifies the corneal deformation, *δ*, under air puff together with the corresponding meridional angle, *α*.

**Figure 3:**
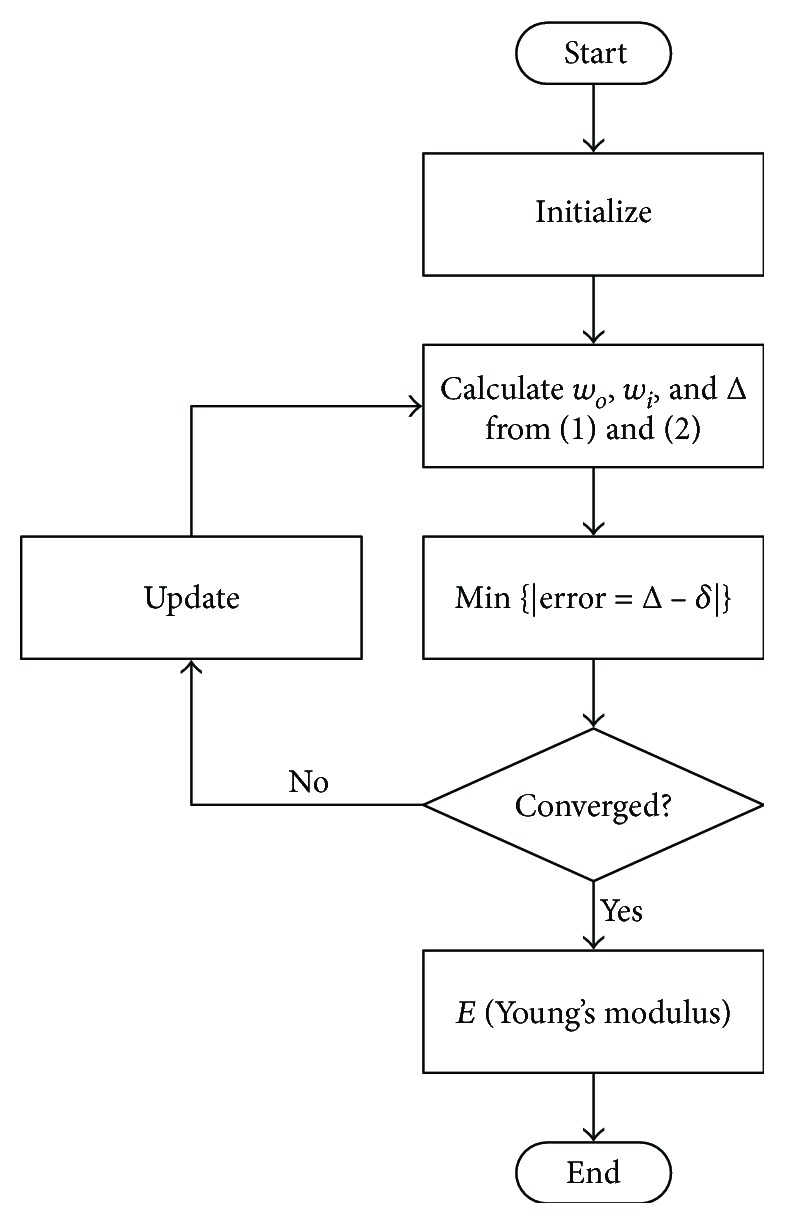
Flowchart for corneal Young's modulus estimation.

The corneal radius *R* can be calculated from *R*^2^ = (*X*^2^ + *Y*^2^)/2*Y*, and the meridional angle *α* is given by *α* = sin^−1^(*X*/*R*). Note that the low-resolution high-speed Scheimpflug imaging-based deduction may introduce additional uncertainties. In this experiment, the pixel size is 0.017 mm which imposes a resolution limit of 0.034 mm (7% of the corneal thickness). That 7% error could contribute to a major disadvantage of the proposed method. In addition, there are still some parameters that has to be determined beforehand. For example, the Poisson's ratio *ν* of the cornea is set to be 0.49; the maximum pressure of the air puff is set to 60 mmHg; and the area of the cord of the maximal deflection is set to *A* = *πR*^2^sin^2^(*α*). A ratio of (2/3)^2^*A* is used to represent the nonuniform pressure distribution over the air puff area [34]. As mentioned in the last section, the Young's modulus is not explicit and is embedded within the normalized force $\bar{p}=pR^{2}/Et^{2}$, which could be obtained from (22) and (23). Since (22) is not a linear equation, it is necessary to use a minimization procedure to search for the optimum solutions of the two unknowns, ${\bar{w}}_{i}$ and ${\bar{w}}_{o}$. The search is carried out to minimize the error between the corneal displacement Δ calculated from the mathematical model in (23) and the corneal deflection *δ* by the Corvis ST measurement. In practice, the minimization of error = |Δ − *δ*| is executed by applying the numerical SQP method (Sequential Quadratic Programming) [39]. With the knowledge of ${\bar{w}}_{i}$ and ${\bar{w}}_{o}$, it is now straightforward to substitute for the corneal Young's modulus. The procedure is illustrated in Figure 3.

### 2.4. Validation and Statistical Analysis

We retrospectively enrolled the images from 536 right eyes of 536 healthy subjects (228 male and 308 female) at the Department of Ophthalmology, National Taiwan University Hospital, and Hung-Chuo Eye Clinics between March of 2012 and April of 2016. The research was approved by the Ethical Review Board of the National Taiwan University. The content of the research followed the tenets of the Declaration of Helsinki. The measurement was performed as previously described [40–42]. Briefly, the recording started with the cornea at the natural convex shape. The air puff would push the cornea in through the first applanation until it reaches a maximum convex inward, referred to as the highest concavity (HC). There would then be a slight oscillation before the cornea bounced back through the second applanation and restored to its natural shape. A1T marked the time it took to reach the first applanation. A1L and A1V were the corresponding length (diameter) of the flattened cornea and the velocity of the movement. Likewise, A2T marked the time stamp for the second applanation, and A2L and A2V represented the diameter and the velocity of the cornea at the second applanation. During the measurement, the movement of the cornea was compensated by the movement of the whole eye; however, only the movement of the cornea was recorded. The CCT in the measure was taken off the Scheimpflug image and the lowest value was recorded.

To avoid multicollinearity, the correlations between any two variables were analyzed for the right eyes of all subjects by Pearson correlation to study the effect of corneal parameters on the IOP. The more significant variables such as the age, sex, and CCT were considered. The effects of the parameters and the Young's modulus on the measured IOP were analyzed by multivariate linear regression, allowing us to consider data from the correlation coefficients. The multivariate linear regression is based on cases with no missing values for any variables used, and the syntax is in missing listwise, outs ranova, and stepwise (age, CCT, and Young's modulus) modes. The accuracy of our models was confirmed by the goodness-of-fit statistic pseudo R^2^ with a least-square method. The residual sum of squares was estimated as the unexplained proportion of IOP variation.

(26) $$\begin{array}{c} R^{2}=\left(1-\frac{residual\;sum\;of\;squares}{total\;variation}\right)=the\;explained\;proportion\;by\;the\;model. \end{array}$$

All statistical analyses were performed using SAS 9.3 (Cary, NC, USA).

## 3. Results

There were 11 corneal parameters obtained from the Corvis ST, and the Young's moduli of both eyes were analyzed and listed in Tables 1 and 2. Most of the parameters measured from the Corvis ST were not significantly different between the two eyes except the peak distance (PD) (*P* < .001). The mean Young's moduli of the right eyes (0.207 MPa; 95% CI, 0.054–0.359 MPa) and the left eyes (0.205 MPa; 95% CI, 0.070–0.339 MPa) were also very close. All of these parameters including the Young's modulus were not significantly different among these three age groups: 0–14, 15–64, and older than 64 years old (one-way ANOVA, *P* > .05) as shown in Table 1. However, the Young's modulus gradually decreased in the elderly group.  Table 2 showed the correlation coefficients among the 11 corneal parameters obtained from the Corvis ST and the Young's modulus. We found that the Young's modulus was weakly correlated with the other parameters and the IOP. In contrast, a highly negative correlation coefficient between A1T and IOP was noted. We also found that there were moderately negative or positive correlations between the IOP and the A1V, A2V, DA, A2T, and PD as expected (numbers in the parenthesis are the coefficients). Interestingly, the CCT was weakly correlated with the IOP and the Young's modulus. We further used univariate analysis to demonstrate weak correlations between the Young's modulus and age, IOP, CCT, DA, A1T, and spherical equivalence (in Figure S1a–f).

Table 3 shows the effect of these factors on the IOP in a multivariate linear regression model. The significant predictors were CCT (*P* < .0001), age (*P* = .0001), and the Young's modulus (*P* < .0001). A goodness-of-fit *R*^2^ correlation coefficient was 0.1338 for CCT, 0.0104 for the Young's modulus, and 0.02517 for age. As a result, we could derive the prediction model as follows:

(27) $$\begin{array}{c} IOP\left(mmHg\right)=-8.106+0.034\left(age,years\right)+0.036\left(CCT,\mu m\right)+8.922\left({Young}^{\prime }s\;modulus,MPa\right). \end{array}$$

## 4. Discussion

In this paper, we propose a simplified closed-form solution for a quick estimation of the corneal biomechanical properties during IOP measurement by using the Corvis ST. The model is based upon the nonlinear static fluid-filled hemispherical shell model subjected to a concentrated load. The proposed model is easy to implement and can directly provide the Young's modulus as opposed to the various parameters defined by the ORA or the Corvis ST. A database of the Young's moduli and the damping ratios of 536 subjects' eyeballs was established by fitting the model into experiments. The average Young's modulus found in our study is 0.207 MPa (95% CI, 0.054–0.359 MPa), which is in agreement with other current studies. Our approach improved from the aforementioned methods in that we measured the actual corneal deformations, used the displacements from the Scheimpflug images of the Corvis ST, and presented *in vivo* values of the corneal Young's moduli. The Young's modulus from the proposed method is independent of the IOP, CCT, and other geometrical parameters. This is to be expected because it represents the corneal mechanical property. Because the factors involved in the calculation in this method were all derived from clinical examinations, and the corneal images were derived from the actual deformations, we believe the implementation is clinically feasible and is compatible with our daily practice.

The Young's modulus is a material property that is not supposed to be dependent on geometry or external forces. The proposed model uses only the geometry of the deformed cornea under the impinging air puff. With Scheimpflug imaging, the automated process identifies the meridional angle and derives all the necessary information from the deformation induced by the impinging air puff applying on the surface area of the cornea, which indents and deforms the cornea. Subsequently, in the Corvis ST, DA and PD are measurements of the deformation and are used to derive the corresponding strain by normalizing the circumference length or radius. Accordingly, it is reasonable to expect that the Young's modulus from the proposed method is independent of the IOP, CCT, and other geometrical parameters. However, the statistical analysis still showed a weak correlation between the deduced Young's modulus with the radius, CCT, and PD, as shown in Table 2. Most importantly, the deduced Young's modulus is weakly and negatively correlated with the CCT and indicates that a thin cornea leads to slightly higher Young's modulus. To explain this observation, we reason that the stiffer layers, the Bowman's layer, and the Descemet's membrane [43] contribute more to the overall corneal Young's modulus in a relatively thinner cornea. Thus, a thinner cornea could show a slightly large Young's modulus. Moreover, the radius, CCT, and PD are measured from Scheimpflug images by counting pixels within dozens of micrometers, which may lead to poor resolution and, thus, a larger error in the current experiment.

There was a wide range of Young's moduli, 0.10–110.32 MPa, measured from ex vivo human corneas reported in the literature. In comparison with the *in vivo* literatures, there were few results possibly due to the difficulty in the measurement technique. Hamilton et al. reported the *in vivo* Young's moduli, 0.29 ± 0.06 MPa (95% CI, 0.17–0.40 MPa), by applying the Orssengo-Pye model integrated with the CCT and the IOP measured from the Goldmann applanation tonometer [44]. Lam et al. used a corneal indentation device to measure the corneal stiffness and the tangent elastic modulus, which was 0.755 ± 0.159 MPa after being normalized to a normal IOP of 15.5 mmHg [45]. They derived their results from five independent parameters: CCT, Poisson's ratio, radius, the geometry constant, and the corneal stiffness. Unfortunately, their corneal stiffness included structural stiffness and material stiffness and was not an independent parameter. As a remedy, they introduced other geometrical constants to modify their corneal stiffness.

Compared with other corneal biomechanical properties, the Young's modulus [46] is a well-known physical property that provides a more direct measure for the ability of a substance to resist elastic deformation than the common indices used clinically. As we know, the cornea is a viscoelastic material [47] that returns to its original shape after the load such as the applanation created by an air puff is removed. Vito et al. and Lanza et al. stated that the corneal tissue behaves as a nearly incompressible, linear elastic, homogeneous, isotropic material undergoing a small deformation [48, 49]. Therefore, a constant Young's modulus is expected in the corneal tissue with linear elastic properties. In contrast, for normal individuals, the CH has been shown to have a moderate correlation with IOP and CCT [50, 51]. Lau and Pye reported that both CH and CRF (ORA) were associated with CCT (*R*^2^ = 0.252 and *R*^2^ = 0.290, resp.) [52]. Our tests, on the other hand, showed that the explained proportion *R*^2^ is 0.0273 by the Young's modulus and CCT, a strong indication that the corneal Young's modulus is independent of the corneal volume or its thickness. Kotecha et al. described an IOP-independent biomechanical property of the cornea (corneal constant factor) using the ORA that is calculated as [P1 − (P2/1.27)] [53]. However, this factor increased with thicker CCT and decreased with greater age: = [(0.036 × CCT) − (0.028 × age)] + 1.06 (adjusted *R*^2^ = 0.34; *P* < 0.0001 for CCT, *P* = 0.007 for age). Similarly, in a Chinese population, corneal curvature and axial length were reported to be influencing factors of CH and CRF [54]. Hon and Lam showed that the DA was negatively correlated with CCT (*r* = −0.53, *P* < 0.001) but not with corneal curvatures (flattest curvature, *r* = 0.13, *P* = 0.46; steepest curvature, *r* = 0.05, *P* = 0.75) [19]. They even concluded that a thinner cornea was associated with a higher corneal deformation and that a measurement of DA could serve as an indicator of corneal biomechanical properties. We reason that the CH and the CRF are related to force balance involving the stiffness of the corneal arch structure. The DA and A1T from the Corvis ST are relative to the geometric displacement, which contains corneal mass inertia. Therefore, these parameters would be affected by the other geometrical properties, such as CCT, and were not independent parameters.

There are still some limitations in our model. Our model is derived from the Taber model and applied for the corneal force equilibrium, and it considers three deformation parts: dimple deflection, stretching, and bending. The rough estimation of dimple deflection and stretching reaches 90% of the deformed energy of the whole system. Thus, the simplified model in this paper neglects bending to yield a simplified solution for the force equilibrium of the cornea, but there are three limitations to this approach. The first limitation is relative to the bending aspect. The angle between the upper shell and the lower shell is 2*α* as shown in Figure 1, and this angle is limited to be lower than *π*/3. For a normal cornea, the angle at the first applanation is around *π*/6, and the angle at the maximum deformation for the Corvis ST is less than *π*/4. If this angle is larger than *π*/3, the neglected deformation energy could be underestimated. The second limitation is that the thickness of the cornea is assumed to be uniform in this model. The thickness of the deformed cornea is very similar, and thus we assume the CCT as the average thickness of the deformed area. The third limitation is that (22) is satisfied under the small deformation assumption. Therefore, the force balance between the internal pressure and the external force is perfectly satisfied. Otherwise, the internal pressure (or the IOP) would be underestimated. Taking a closer look at the mechanical properties, the IOP and the Young's modulus could still be affected by the intraocular pressure stretching the cornea. The Young's modulus is affected by stress hardening. To account for this effect, Rayleigh introduced the effective Young's modulus, *E'*, in which a tension term *T* was added to the Young's modulus. According to Rayleigh, *E*′ = *E* + *T*, in which *T* = *pR*/2*t*. Accordingly, if we say the intraocular pressure *P* = 15 mmHg, *R* = 0.7 cm, and *t* = 550 *μ*m, the modification term *T* = 0.013 MPa, accounting for 6.27% of the effective Young's modulus (the effective corneal Young's modulus averaged, *E*′ = 0.207 MPa). If we consider the added term *T* in Figure S1b, we find the unadjusted *R*^2^ is reduced from 0.0104 to 0.00435, and the IOP affects the effective Young's modulus by 7 ± 2%. To conclude, this shows that the Young's modulus is even less dependent on the IOP. It is also noted that table shows a slight decrease in the Young's modulus in the elderly age group. A result that is in contrast with the increasing trend reported in [55]. This is both the strength and limitation of the proposed method. The cornea is an anisotropic material with its fiber families orientated parallel to the corneal surface [56]. This arrangement strengthens the tensile stiffness rather than the bending stiffness. The cross-sectional area of the fiber that increases with age [57] also resulted in enhancing of the tensional stiffness. Elsheikh et al. [55] used the intact cornea subjected to posterior inflation pressure to test the tensional modulus of the corneal tissues. In addition, the additional nonenzymatic cross-linking that occurs with age also strengthens the stromal collagen fibrils. This study, on the other hand, used the maximum deformation amplitude' (DA) from the air puff which included the stiffness induced by the bending effect. The bending strength depends not only on the fiber strength but also on the area moment of inertia of the cornea which is a geometrical property used in the calculation of the deflection. The area moment of inertia contained the integration of the squared distance over the area, *I* = ∬*x*^2^*dA*. The interfibrillar distance decreases with age [57] leading to fast decrease of the bending stiffness. As a result, the calculation showed that the Young's modulus decreases slightly with increasing age.

## 5. Conclusions

This paper proposed a simplified closed-form solution for a quick estimation of the corneal biomechanical properties during IOP measurement by using the Corvis ST. The average Young's modulus of 536 subjects found in our study is 0.207 MPa (95% CI, 0.054–0.359 MPa), which is in agreement with other current studies. The Young's modulus in this model was treated as an independent parameter to represent the mechanical stiffness of the cornea. The Young's moduli were proven to be similar between the two eyes and would decrease slightly in the elderly group. The statistics also showed that the Young's moduli correlated weakly with age, IOP, CCT, DA, and A1T. The proposed method is based on solid mathematical background. The fact that the method directly treated the Young's modulus as an independent parameter and the result that it was only weakly correlated with the rest of the tonometer parameters is in good agreement with the common perception of a mechanical property. This is a major advantage of the proposed method over the other Scheimpflug imaging methods that often introduced individually defined indices. On the other hand, the measurement resolution in this experiment is limited by the low-resolution high-speed camera. This resulted in a 7% measurement uncertainty. To conclude, the proposed approach enables independent measurement of the human corneal mechanical properties *in vivo* and can help quantify clinical indications.

## Supplementary Material

Table S1. Young's moduli collected from previous literature. Table S2. Corvis' parameters and the proposed Young's modulus from right and left eyes of 536 subjects. Figure S1a. The relationship between the Young's modulus and CCT. Figure S1b. The relationship between the Young's modulus and IOP. Figure S1c. The relationship between the Young's modulus and DA. Figure S1d. The relationship between the Young's modulus and A1T. Figure S1e. The relationship between the Young's modulus and age. Figure S1f. The relationship between the Young's modulus and spherical equivalent.

## Figures and Tables

**Table 1:** Corvis' parameters and the proposed Young's modulus from right eyes of 536 subjects in the three different age groups.

Age (years)	0–14	15–64	> 64
	Mean (95% CI)	Mean (95% CI)	Mean (95% CI)
Number	13	498	25
IOP (mmHg)	15.19 (8.72, 21.67)	14.73 (7.73, 21.72)	17.66 (9.16, 26.16)
A1L (mm)	1.78 (1.63, 1.93)	1.77 (1.54, 2.00)	1.81 (1.70, 1.93)
A1V (m/s)	0.14 (0.09, 0.18)	0.15 (0.10, 0.19)	0.12 (0.07, 0.17)
A2L (mm)	1.73 (1.10, 2.37)	1.72 (1.07, 2.37)	1.81 (1.39, 2.22)
A2V (m/s)	−0.35 (−0.51, −0.18)	−0.37 (−0.55, −0.20)	−0.32 (−0.45, −0.19)
PD (mm)	3.91 (1.23, 6.5)	3.79 (1.22, 6.36)	3.56 (1.02, 6.09)
Radius (mm)	7.11 (5.01, 9.20)	7.17 (4.72, 9.63)	7.63 (5.05, 10.21)
DA (mm)	1.02 (0.75, 1.29)	1.07 (0.83, 1.31)	1.03 (0.81, 1.25)
CCT (*μ*m)	537.54 (482.52, 592.56)	543.10 (469.31, 616.90)	561.68 (484.96, 638.40)
A1T (msec)	7.45 (6.63, 8.27)	7.38 (6.53, 8.23)	7.76 (6.73, 8.79)
A2T (msec)	21.76 (20.60, 22.93)	21.77 (20.77, 22.77)	21.21 (20.26, 22.16)
*E* (MPa)	0.243 (0.089, 0.397)	0.208 (0.053, 0.363)	0.187 (0.104, 0.269)

A1T and A2T: applanation times; A1L and A2L: applanation diameters; A1V and A2V: applanation velocities; PD: peak distance; DA: maximum deformation amplitude; CCT: central corneal thickness; *E*: Young's modulus.

**Table 2:** Pearson correlation coefficients between different parameters of the Corvis ST measurement and the proposed Young's modulus.

	IOP	A1L	A1V	A2L	A2V	Radius	DA	CCT	A1T	A2T	PD	*E*
IOP	1											
A1L	.219^∗∗^	1										
A1V	**−.590** ^∗∗^	−.072	1									
A2L	.190^∗∗^	.229^∗∗^	−.169^∗∗^	1								
A2V	**.612** ^∗∗^	.153^∗∗^	−.493^∗∗^	.269^∗∗^	1							
Radius	.297^∗∗^	.198^∗∗^	−.239^∗∗^	.194^∗∗^	.366^∗∗^	1						
DA	**−.778** ^∗∗^	−.162^∗∗^	**.597** ^∗∗^	−.200^∗∗^	**−.692** ^∗∗^	−.402^∗∗^	1					
CCT	.366^∗∗^	.315^∗∗^	−.262^∗∗^	.280^∗∗^	.425^∗∗^	.353^∗∗^	−.318^∗∗^	1				
A1T	**.986** ^∗∗^	.216^∗∗^	**−.600** ^∗∗^	.197^∗∗^	**.617** ^∗∗^	.299^∗∗^	**−.775** ^∗∗^	.371^∗∗^	1			
A2T	**−.794** ^∗∗^	−.190^∗∗^	**.604** ^∗∗^	−.097^∗^	**−.551** ^∗∗^	−.295^∗∗^	**.735** ^∗∗^	−.307^∗∗^	**−.808** ^∗∗^	1		
PD	**−.737** ^∗∗^	−.098^∗^	.487^∗∗^	−.164^∗∗^	**−.677** ^∗∗^	−.212^∗∗^	**.750** ^∗∗^	−.299^∗∗^	**−.732** ^∗∗^	**.630** ^∗∗^	1	
*E*	.102^∗^	−.042	−.181^∗∗^	−.070	.054	−.250^∗∗^	−.097^∗^	−.165^∗∗^	.108^∗^	−.087^∗^	−.201^∗∗^	1

^∗∗^Correlation is significant at the 0.01 level (2-tailed); ^∗^Correlation is significant at the 0.05 level (2-tailed). A1T and A2T: applanation times; A1L and A2L: applanation diameters; A1V and A2V: applanation velocities; PD peak distance; DA: maximum deformation amplitude; CCT: central corneal thickness; *E*: Young's modulus.

**Table 3:** Association of parameters with measured IOP using a multiple regression model.

	Multiple linear regression		
Variable	Estimate	*S.E.*	*P*
Intercept	−8.106	2.208	0.000
Age	.034	.010	0.001
CCT	.036	.004	0.000
Young's modulus	8.922	1.894	0.000
