# Supplementary material for: Estimation of the Corneal Young's Modulus In Vivo Based on a Fluid-Filled Spherical-Shell Model with Scheimpflug Imaging
Source: J Ophthalmol. 2017 Nov 8;2017:5410143. doi: 10.1155/2017/5410143 (PMC5698833; doi:10.1155/2017/5410143)
Supplement: Supplementary file 1 — Table S1. Young's moduli collected from previous literature. Table S2. Corvis' parameters and the proposed Young's modulus from right and left eyes of 536 subjects. Figure S1a. The relationship between the Young's modulus and CCT. Figure S1b. The relationship between the Young's modulus and IOP. Figure S1c. The relationship between the Young's modulus and DA. Figure S1d. The relationship between the Young's modulus and A1T. Figure S1e. The relationship between the Young's modulus and age. Figure S1f. The relationship between the Young's modulus and spherical equivalent. [file 5410143.f1.pdf]

SUPPLEMENTARY MATERIALS

There were a wide range of Young's moduli, 0.10–110.32 MPa, measured from *ex vivo* human corneas reported in the literature, as shown in Table S1. Glass et al. applied a three-component spring and dashpot model to match the dynamic response with the kinematic information deduced from the high-speed camera images. They compared the modeling results with corneal phantom experiments to demonstrate how viscosity and elasticity can change the corneal hysteresis. People also tried to measure the Young's moduli of the different layers of the cornea tissue by using the atomic force microscope (AFM). A flying spot scanner [1], the measurement of mercury drop displacement in a whole eye under increasing pressure [2], an ultrasonic technique [3], a pressure system used to examine quasi-static and dynamic rupture pressure [4], a stress-relaxation test in a vertical corneal strip[5], a radial shearing speckle pattern interferometer after an increase in intraocular pressure from 15.0 to 15.5 mmHg [6], a Scheimpflug corneal three-dimensional topographer for stress-strain curves while IOP either remained constant or increased by 40 mmHg and then decreased (at 4-mmHg steps)[7], an atomic force microscope [8, 9], a transverse biaxial resistance test [10], Scheimpflug topography [11], and acoustic radiation force elasticity microscopy [12]. Despite the improvements in the measurement methods, there was still a wide variation in *ex vivo* measurement of the Young's moduli due to the different methods of measurement, different *ex vivo* preparations, and different storage conditions of the corneas. Furthermore, the cornea is physiologically constrained in an orbital socket, covered by a tear film layer and a thick eyelid that blinks frequently and

remains at a relatively constant temperature; accordingly, the *in vivo* cornea in the eye socket should differ from an enucleated eyeball or an extracted corneal strip and would present different biomechanical properties.

TABLE S1. Young's moduli collected from previous literature

No	Reference	Years	Young's modulus	Methods	*ex vivo* or *in vivo*
**1**	[1]	1972	1.8-8.1 MPa	flying spot scanner	*ex vivo*
**2**	[2]	1986	≈5 MPa	displacements of two very small mercury drops on the corneal surface	*ex vivo*
**3**	[3]	1996	4.2-30 MPa	the mercury drop displacement in a whole eye under increasing pressure	*ex vivo*
**4**	[13]	1999	50-100 MPa	modules of the Hypermesh for solid modeling, geometric construction, and finite element mesh creation	*ex vivo*
**5**	[4]	2003	6.89-110.32 MPa	quasi-static and dynamic rupture pressure	*ex vivo*
**6**	[14]	2005	45. 74± 1. 69 MPa (43.25-48.67 MPa)	one dimension tensile test, tensile stress relaxation and creep test	*ex vivo*
**7**	[5]	2007	0.16-0.81 MPa	an inflation condition to determine pressure-deformation results using shell theory to derive the relationship between the modulus of elasticity and IOP	*ex vivo*
**8**	[15]	2008	0.15-1.15 MPa	inflation test	*ex vivo*
**9**	[7]	2010	0.692 ± 0.30 MPa	Scheimpflug corneal three-dimensional topographer for whole globe stress-strain curves while IOP either remained constant or increased by 40 mm Hg and then decreased (4-mm Hg steps)	*ex vivo*
**10**	[6]	2011	0.27-0.52 MPa	radial shearing speckle pattern interferometer after an increase in intraocular pressure from 15.0 to 15.5 mm Hg	*ex vivo*
**11**	[16]	2012	1.14 and 2.63 MPa	atomic force microscope, and it was constant over the range of indentation depths between 1.0 and 2.7 μm in the stroma	*ex vivo*
**12**	[17]	2012	7.5 ± 4.2 kPa (anterior basement membrane), 109.8 ± 13.2 kPa (Bowman's layer), 33.1 ± 6.1 kPa(anterior stroma),	atomic force microscope	*ex vivo*

			50 ± 17.8 kPa (Descemet’s membrane).		
**11**	[9]	2013	1.48 ± 0.17 to 10.19 ± 1.06 MPa	atomic force microscope	*ex vivo*
**12**	[12]	2014	39 ± 0.28 kPa for the central anterior cornea 0.71 ± 0.21 kPa for the central posterior cornea	acoustic radiation force elasticity microscope	*ex vivo*
**13**	[11]	2014	2.28 ± 0.87 and 3.30 ± 0.90 at the anterior cornea, 0.21 ± 0.09 and 0.17 ± 0.06 at the posterior cornea with and without intact epithelium	Scheimpflug topography	*ex vivo*
**14**	[8]	2015	0.2459 ± 0.2091 (82.3 - 530.8 kPa) for the anterior cornea, 0.1002 ± 0.0619 (range: 28.1 - 162.6 kPa) for the posterior cornea	atomic force microscope	*ex vivo*
**15**	[10]	2015	3.7 ± 2.5 MPa at 10% strain, 9.5 ± 1.8 MPa at 20% strain	transverse biaxial resistance	*ex vivo*
**16**	[18]	2008	0.13-0.43	applanation tonometer	*in vivo*
**17**	[19]	2015	0.755 ± 0.159 MPa	corneal indentation device; tangent elastic modulus; after being normalized to normal intraocular pressure of 15.5 mmHg	*in vivo*

Table S2. Corvis' parameters and the proposed Young's modulus from right and left eyes of 536 subjects.

	Right eye<br>Mean (95% CI)	Left eye<br>Mean (95% CI)	t-test<br>(P value)
IOP (mmHg)	14.87 (7.7-22.05)	14.80 (7.69, 22.27)	0.642
A1L (mm)	1.77 (1.55, 2.00)	1.78 (1.58, 1.98)	0.545
A1V (m/s)	0.14 (0.10, 0.19)	0.14 (0.10, 0.19)	0.585
A2L (mm)	1.72 (0.91, 2.56)	1.72 (1.07, 2.36)	0.702
A2V (m/s)	-0.37 (-0.54, -.20)	-0.37 (-0.55, -0.20)	0.649
PD (mm)	3.77 (1.20, 6.35)	4.07 (1.54, 6.60)	<0.001
Radius (mm)	7.19 (4.74, 9.65)	7.22 (4.83, 9.61)	0.847
DA(mm)	1.06 (0.82, 1.31)	1.07 (0.82, 1.32)	0.649
CCT (μm)	543.87 (469.86, 617.82)	542.80 (469.62, 615.97)	0.978
A1T (msec)	7.40 (6.52, 8.27)	7.40 (6.49, 8.30)	0.811
A2T (msec)	21.74 (20.42, 23.00)	21.75 (20.46, 23.02)	0.343
E (MPa)	0.207 (0.054, 0.359)	0.205 (0.070, 0.339)	0.524

A1T and A2T: A time-1/-2; A1L and A2L: A length-1/-2; A1V and A2V: velocity-1/-2; PD peak distance; DA: maximum deformation amplitude; CCT: central corneal thickness; *E*: Young's modulus.

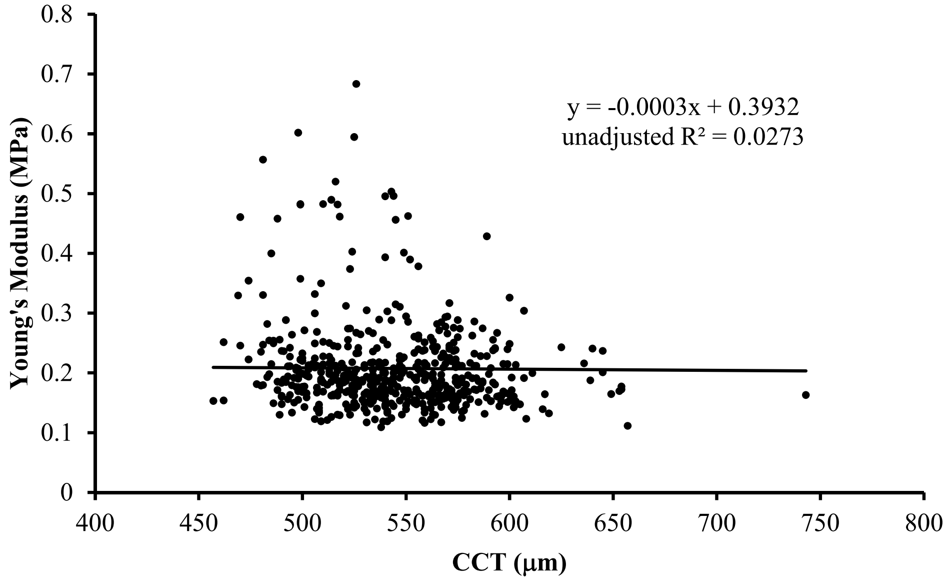

Figure S1a. The relationship between the Young's modulus and CCT.

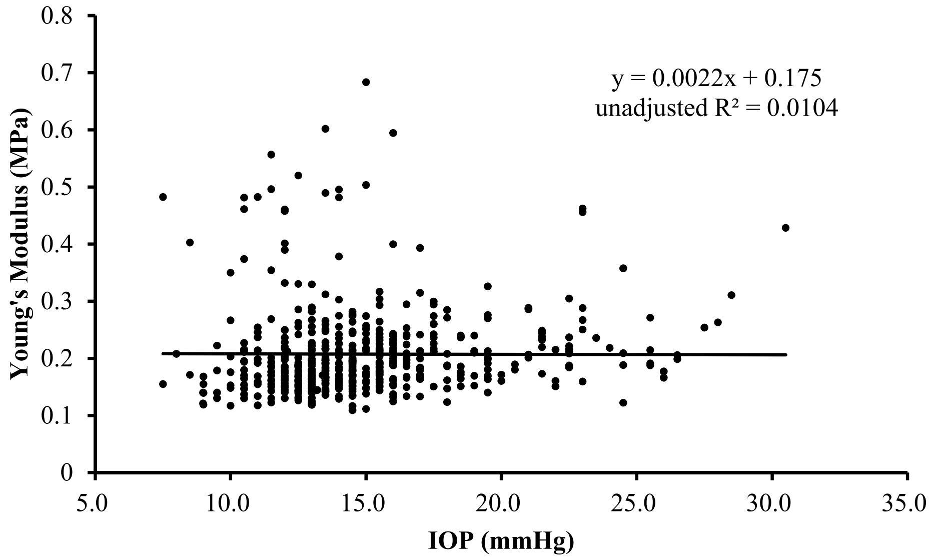

Figure S1b. The relationship between the Young's modulus and IOP.

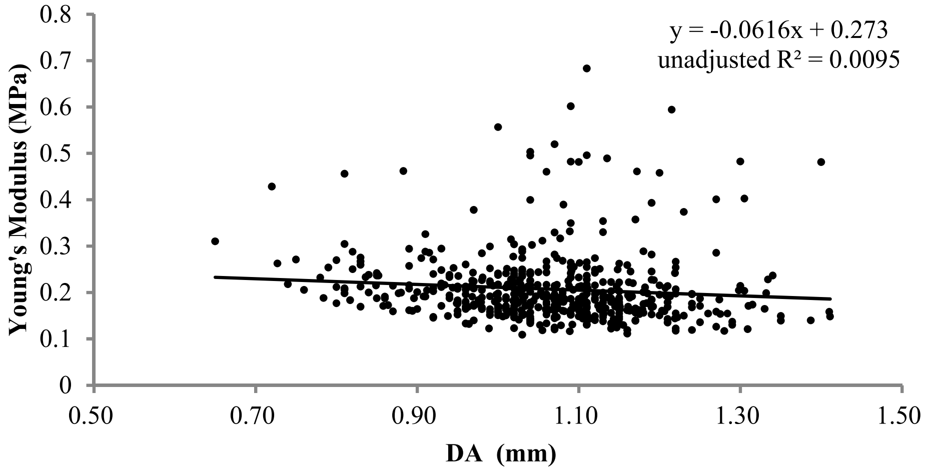

Figure S1c. The relationship between the Young's modulus and DA.

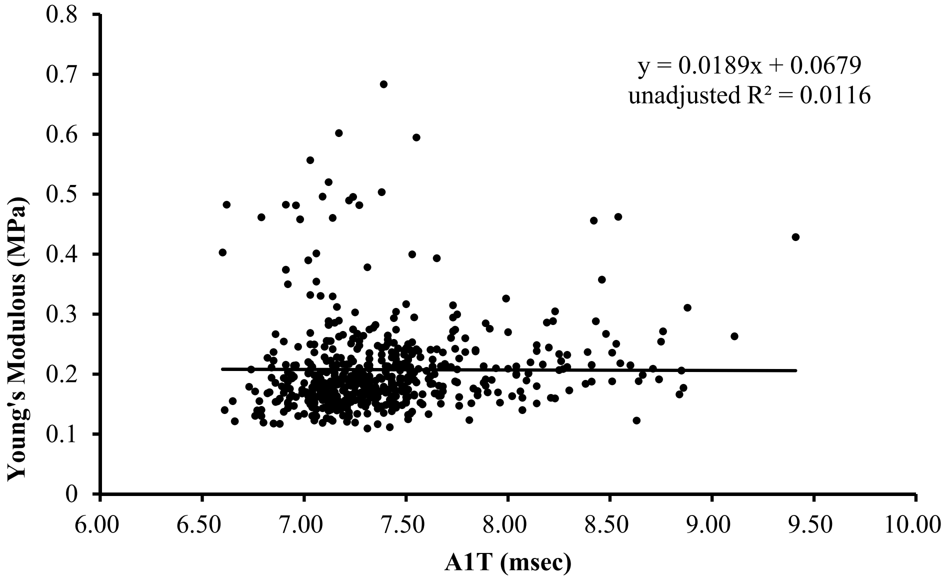

Figure S1d. The relationship between the Young's modulus and A1T.

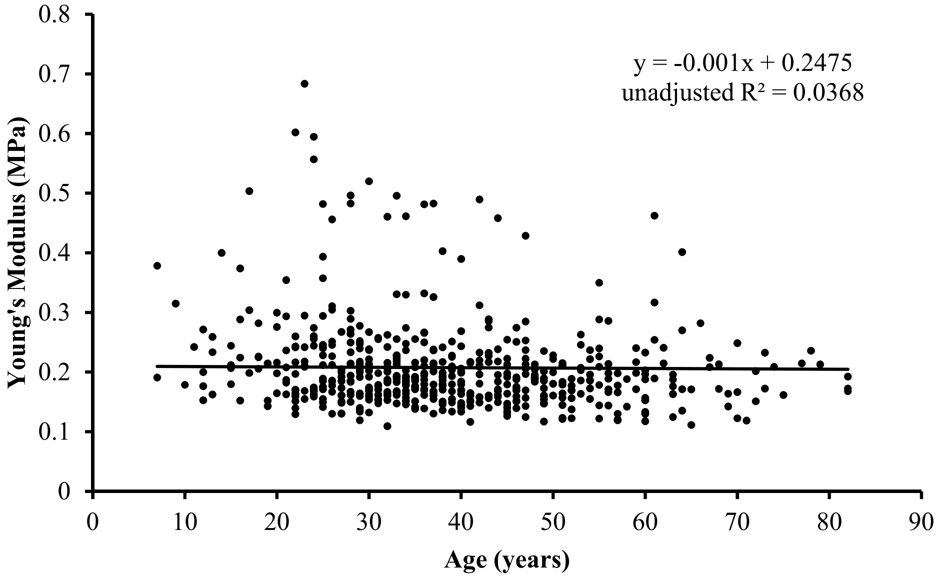

Figure S1e. The relationship between the Young's modulus and age.

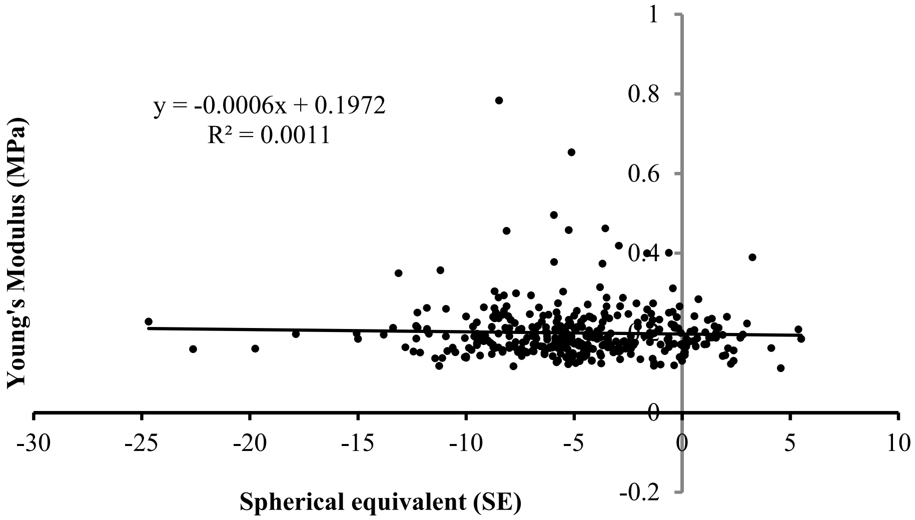

Figure S1f. The relationship between the Young's modulus and spherical equivalent.

1. Woo, S.L.Y., et al., *Nonlinear Material Properties of Intact Cornea and Sclera.* Experimental Eye Research, 1972. **14**(1): p. 29-&.

2. Jue, B. and D.M. Maurice, *THE MECHANICAL-PROPERTIES OF THE RABBIT AND HUMAN CORNEA.* Journal of Biomechanics, 1986. **19**(10): p. 847-853.

3. Wang, H., et al., *An ultrasonic technique for the measurement of the elastic moduli of human cornea.* J Biomech, 1996. **29**(12): p. 1633-6.

4. Voorhies, K.D., *Static and dynamic stress/strain properties for human and procine eyes*, in *Mechanical Engineering*. 2003, Virginia Polytechnic Institute and State University: Blacksburg, VA, USA.

5. Elsheikh, A., D.F. Wang, and D. Pye, *Determination of the modulus of elasticity of the human cornea.* Journal of Refractive Surgery, 2007. **23**(8): p. 808-818.

6. Knox Cartwright, N.E., J.R. Tyrer, and J. Marshall, *Age-related differences in the elasticity of the human cornea.* Invest Ophthalmol Vis Sci, 2011. **52**(7): p. 4324-9.

7. Kling, S., et al., *Corneal biomechanical changes after collagen cross-linking from porcine eye inflation experiments.* Invest Ophthalmol Vis Sci, 2010. **51**(8): p. 3961-8.

8. Labate, C., et al., *Understanding of the viscoelastic response of the human corneal stroma induced by riboflavin/UV-a cross-linking at the nano level.* PLoS One, 2015. **10**(4): p. e0122868.

9. Dias, J., et al., *Anterior and posterior corneal stroma elasticity after corneal collagen crosslinking treatment.* Exp Eye Res, 2013. **116**: p. 58-62.

10. Kanellopoulos, A.J., et al., *High-irradiance CXL combined with myopic LASIK: flap and residual stroma biomechanical properties studied ex-vivo.* Br J Ophthalmol, 2015. **99**(6): p. 870-4.

11. Lombardo, G., et al., *Analysis of the viscoelastic properties of the human cornea using Scheimpflug imaging in inflation experiment of eye globes.* PLoS One, 2014. **9**(11): p. e112169.

12. Mikula, E., et al., *Measurement of corneal elasticity with an acoustic radiation force elasticity microscope.* Ultrasound Med Biol, 2014. **40**(7): p. 1671-9.

13. Uchio, E., et al., *Simulation model of an eyeball based on finite element analysis on a supercomputer.* British Journal of Ophthalmology, 1999. **83**(10): p. 1106-1111.

14. Zhao, M., et al., *[Experimental study on viscoelastic properties of human cornea].* Sheng Wu Yi Xue Gong Cheng Xue Za Zhi, 2005. **22**(3): p. 550-4.

15. Elsheikh, A., D. Alhasso, and P. Rama, *Biomechanical properties of human and porcine corneas.* Exp Eye Res, 2008. **86**(5): p. 783-90.

16. Lombardo, M., et al., *Biomechanics of the Anterior Human Corneal Tissue Investigated with Atomic Force Microscopy.* Investigative Ophthalmology & Visual Science, 2012. **53**(2): p. 1050-1057.

17. Last, J.A., et al., *Compliance profile of the human cornea as measured by atomic force microscopy.* Micron, 2012. **43**(12): p. 1293-1298.

18. Hamilton, K.E. and D.C. Pye, *Young's modulus in normal corneas and the effect on applanation tonometry.* Optom Vis Sci, 2008. **85**(6): p. 445-50.

19. Lam, A.K., et al., *Repeatability of a novel corneal indentation device for corneal biomechanical measurement.* Ophthalmic Physiol Opt, 2015. **35**(4): p. 455-61.
